# Comparing management strategies for conserving communities of climate-threatened species with a stochastic metacommunity model

**DOI:** 10.1098/rstb.2021.0380

**Published:** 2022-08-15

**Authors:** Gregory A. Backus, Yansong Huang, Marissa L. Baskett

**Affiliations:** ^1^ Environmental Science and Policy, University of California, Davis, CA, USA; ^2^ Spanish Institute of Oceanography, Oceanographic Center of the Balearic Islands, Palma de Mallorca, Illes Balears, Spain

**Keywords:** connectivity, assisted migration, restoration, climate change, metacommunity

## Abstract

Many species are shifting their ranges to keep pace with climate change, but habitat fragmentation and limited dispersal could impede these range shifts. In the case of climate-vulnerable foundation species such as tropical reef corals and temperate forest trees, such limitations might put entire communities at risk of extinction. Restoring connectivity through corridors, stepping-stones or enhanced quality of existing patches could prevent the extinction of several species, but dispersal-limited species might not benefit if other species block their dispersal. Alternatively, managers might relocate vulnerable species between habitats through assisted migration, but this is generally a species-by-species approach. To evaluate the relative efficacy of these strategies, we simulated the climate-tracking of species in randomized competitive metacommunities with alternative management interventions. We found that corridors and assisted migration were the most effective strategies at reducing extinction. Assisted migration was especially effective at reducing the extinction likelihood for short-dispersing species, but it often required moving several species repeatedly. Assisted migration was more effective at reducing extinction in environments with higher stochasticity, and corridors were more effective at reducing extinction in environments with lower stochasticity. We discuss the application of these approaches to an array of systems ranging from tropical corals to temperate forests.

This article is part of the theme issue ‘Ecological complexity and the biosphere: the next 30 years’.

## 1. Introduction

The projected rate of climate change threatens many species, especially dispersal-limited species [1]. Habitat fragmentation intensifies this risk by causing the additional impediment of needing to disperse over poor-quality habitat [2]. Moreover, when competing species track climate change at differential speeds, faster-dispersing species can block slower-dispersing species from tracking climate change [3]. Such impediments can have ecosystem-wide consequences when dispersal-limited species serve as foundation species, such as in forests [4] and tropical coral reefs [5]. Though many coral reef species can disperse far in their larval stage, differential dispersal ability and fragmentation could mean that some species are unable to keep pace with climate change [5,6]. Similarly, competition and the differential effects of climate change on tree species mean that poleward species might prevent equatorward species from tracking climate change, especially over fragmented landscapes [7].

One potential method of conserving dispersal-limited species is through assisted migration, or the relocation of populations outside the species' historical range to areas that will be more suitable in response to climate change [8,9]. Despite a long history of conservation translocations within a species’ historical range [10], relocating a species to a new area with novel species interactions could pose additional challenges. With little precedent and high uncertainty, relocated populations could become invasive or spread diseases and parasites [11,12]. Even translocations within a species' range are often unsuccessful without the additional complications of novel competitors, climate change and fragmentation [13,14]. To limit relocation failure, decision-making frameworks for assisted migration generally seek to understand the uncertainty around the optimal time and place to move a vulnerable species [15]. However, assisted migration might have limited success when relocating species with narrow climate tolerance into environments with high climate variability over time or low climate variability over space. Additionally, assisted migration is often a single-species approach [16] that addresses the symptoms of extinction risk instead of the root causes (e.g. habitat fragmentation; [17,18]). Despite potential risks and uncertainties, assisted migration is already underway for several species at risk of extinction, with some variation being tested in coral reefs [19,20] and trees [21].

Alternatively, habitat restoration in and between fragmented habitats could assist the natural dispersal of species that would otherwise be unable to track climate change [16]. Building habitat corridors [22,23] or stepping-stone reserves [24,25] might help increase connectivity and decrease extinction risk from climate change [26], and additional protection of existing reserves might bolster source populations to increase overall persistence [27]. Unlike the single-species focus of assisted migration, increasing habitat protection or connectivity is a community-level approach that could directly benefit multiple species that might otherwise be unable to disperse between fragmented patches [16]. However, increasing connectivity and habitat protection do not specifically target species disproportionally affected by climate change, where biological limitations in dispersal ability and negative effects of community interactions could prevent climate tracking [3,28]. Among the restoration options, those that increase connectivity inherently increase available habitat area, which could be critical for declining populations at risk of extinction from climate change [29]. While increasing connectivity typically has a smaller effect on population outcomes than increasing protection or patch size, or reducing overall habitat loss, in conservation generally [30–32], increasing connectivity might have a greater impact when considering range shift dynamics under climate change [33]. Like assisted migration, the effectiveness of connectivity and restoration-based approaches at conserving species can depend on spatio-temporal variability, as stochasticity in connectivity can reduce species’ persistence [34] while heterogeneity in microclimates can increase persistence through climate change [35]. As an example of a connectivity-based approach, protecting a marine reserve network focused on connectivity between locations with different levels of temperature stress is one proposed approach to buffer coral reef response to climate change [36,37]. For forest trees, connectivity and restoration would involve creating large-scale networks of land-sharing or land-sparing between disconnected forests [38] or working with local landowners to encourage practices that reduce barriers and promote species persistence [2].

Given the potential trade-offs to each approach, we compare the relative efficacy of these alternative management strategies to support species responses to climate change. To understand how these strategies compare under a variety of conditions in terms of spatio-temporal climate variability, we extend a metacommunity model ([39], where the previous analysis focused solely on the management strategy of assisted migration) that simulates climate tracking of several randomized species competing in a fragmented environment over a temperature gradient through a cycle of reproduction, dispersal and competition. Using this model, we compared a variety of management strategies to conserve species' persistence and diversity: assisted migration, building habitat corridors, creating stepping-stone reserves and reinforcing areas that currently had high habitat quality.

## 2. Methods

To compare the potential for various conservation strategies to reduce extinction in environments under different spatio-temporal conditions, we modelled metacommunity dynamics of species competing on a one-dimensional linear temperature gradient subjected to climate change. Building on the models by Backus & Baskett [39] and Urban *et al*. [3], all species in this metacommunity compete for the same resources on the same trophic level. Though other ecological interactions can drive species coexistence [40,41], we chose to focus on competition as the central interspecific interaction in our model because of its role in driving range limits [42,43] and range shifts [3,44]. Each species *i* has a discrete population size *n_i_*(*x*, *y*, *t*) that changes with time *t* and space on both the large scale *x* and local scale *y*. All populations cycle through reproduction, dispersal and competition, each with demographic stochasticity. Each species has a unique thermal optimum *ζ_i_*, dispersal distance *γ_i_*, thermal tolerance breadth *σ_i_*, and reproductive strength *ρ_i_*. Because we were interested in comparing assisted migration with management approaches that affect heterogeneity as well as connectivity under climate change, we expanded the model in Backus & Baskett [39] to include variation in temperature on the local scale and variation in habitat quality on the larger scale. The carrying capacity *K*(*x*, *y*) varies over space to represent high- and low-quality habitat. After simulating metacommunity dynamics with climate change, we compared extinction rates under each approach. Then we focused on comparing corridors with assisted migration for different levels of environmental stochasticity and local heterogeneity, and finally we analysed the species characteristics associated with protection by each approach.

### (a) Climate variability and change

We represent local temperature variation across space with the local climate heterogeneity parameter, *H*. Space in this model is a one-dimensional temperature gradient of *L* patches, representing large-scale latitudinal or elevational change [3]. Each patch *x* ∈ *X* has *W* subpatches, representing small-scale variability in microclimates without an explicit spatial structure. Each local subpatch *y* ∈ *Y* temperature has *T*(*x*, *y*, *t*) with a mean patch temperature of $\bar{T}(x,\;t)=\sum_{y=1}^{W}T(x,\;y,\;t)\;$ at time *t*. We set the local climate heterogeneity such that each patch has a standard deviation in local temperatures of

2.1 $$H=\sqrt{\frac{\sum_{y=1}^{Y}{\left(T(x,\;y,\;t)-\;\bar{T}(x,\;t)\right)}^{2}}{W-1}}.$$

Temperature increases linearly over time with environmental stochasticity, *S*, representing the magnitude of interannual variation in temperature across the environment. At the beginning of each time step, all patches simultaneously increase in temperature by an average value of *τ*, with a stochastic component with autocorrelation *κ*, and standard deviation *S* around white noise *ω*(*t*): $\epsilon (t+1)=\kappa \epsilon (t)+\omega (t)\sqrt{1+\kappa^{2}}$, with the square root term to remove the effect of autocorrelation on the variance [45]. Altogether, the annual temperature change in patch *x,* subpatch *y*, is

2.2 T(x, y, t+1)=T(x, y, t)+τ+Sϵ(t).

### (b) Metacommunity dynamics

**Figure 1.**
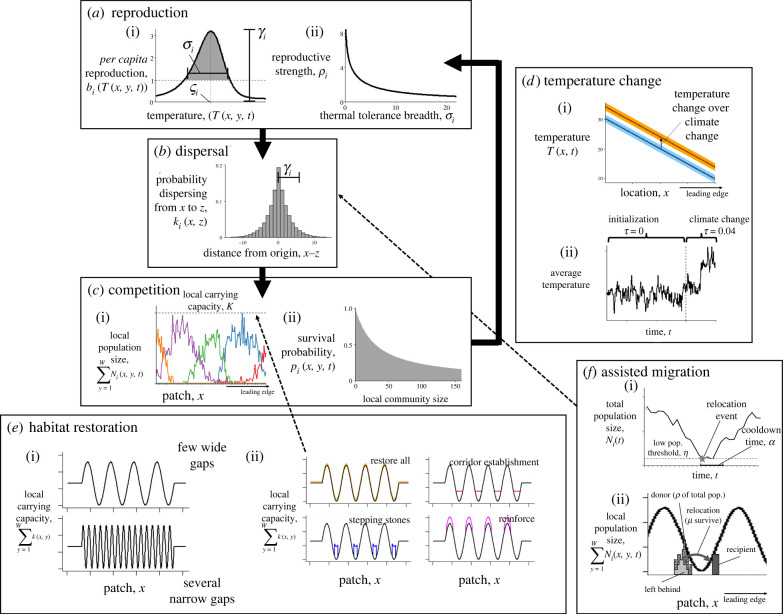
During each time step of the model, all extant species cycle through (*a*) reproduction, (*b*) dispersal and (*c*) competition before (*d*) the temperature changes and the next time step continues. (*a*(i)) *Per capita* reproductive output *b_i_*(*T*(*x*, *y*, *t*)) is skew-normal and dependent on temperature *T*(*x*, *y*, *t*). This function is shaped by species' thermal optimum *ζ_i_* and thermal tolerance breadth *σ_i_*. (*a*(ii)) Reproductive strength *ρ_i_* scales the total reproductive output so that species with narrow *σ_i_* (specialists) have higher reproduction and species with broad *σ_i_* (generalists) have lower reproduction. (*b*) The dispersal kernel is a long-tailed ‘double geometric’ distribution with a mean dispersal distance *γ_i_*. (*c*(i)) All species compete over limited space, where each patch has a carrying capacity *K*(*x*, *y*). Here each line represents a different species. (*c*(ii)) In each patch, individual survival probability *p*(*x*, *y*, *t*) decreases as the total community size increases. (*d*) Temperature changes stochastically over time. (*d*(i)) Mean temperature decreases linearly with space. Over time, between *t* = 0 (lower line) and *t* = 100 (upper line), the temperature increases. (*d*(ii)) Temperature variation over time depends on level of environmental stochasticity. The vertical dashed line designates when the model changes from the initialization phase (average temperature change (*τ* = 0)) to the climate change and intervention phase (*τ* = 0.04). Climate change only occurs after a relatively stable metacommunity has been assembled, after 100 time steps have passed with no extinctions. (*e*(i)) Two types of fragmented environments compared: one with few large gaps and one with several narrow gaps. (*e*(ii)) The four restoration management strategies (coloured lines). Each involved increasing the integral of carrying capacity over space by an amount *E* more than the original carrying capacity (black lines). (*f*(i)) Relocation occurs once the total population of a species falls below a threshold *η*. To avoid repetition while the species recovers, no relocations occur during a cooldown period following relocation *α*. (*f*(ii)) A fraction *ρ* of the population is removed from its original distribution and moved to the closest new location where the average temperature $\bar{T}(x,\;t)\leq \zeta_{i}+0.2$ and the carrying capacity *K*(*x*, *y*) > 5 (only a fraction *μ* survive). Remaining individuals disperse naturally.

Each simulated species *i* has a population size population size of *n_i_*(*x*, *y*, *t*) individuals in patch *x*, subpatch *y* at discrete time *t*. All individuals reproduce simultaneously at the beginning of each time step with a reproductive output *b_i_*(*T*(*x*, *y*, *t*)) as a function of time- and location-dependent temperature (figure 1*a*). Temperature dependence is skew-normal, given skewness constant *λ* with the highest values around the species’ thermal optimum *ζ_i_* and a sharp decrease above *ζ_i_* [46]. Then given, thermal tolerance breadth *σ_i_* and fecundity *ρ_i_*, the reproductive output is

2.3 $$b_{i}(T(x,\;y,\;t))=\exp\left(\rho_{i}\left\{\exp\left[-{\left(\frac{T(x,\;y,\;t)-\zeta_{i}}{\sigma_{i}}\right)}^{2}\right]\times \left[1+\;\mathrm{erf}\left(\lambda \frac{T(x,\;y,\;t)-\zeta_{i}\;\;}{\sigma_{i}}\right)\;\right]-1\right\}\right)$$

[3]. In parameterizing our model (as described in §2d, Numerical implementation) we assume a trade-off between thermal tolerance breadth and fecundity. To incorporate demographic stochasticity, the number of propagules produced by individuals in patch *x*, subpatch *y* is a Poisson random variable with mean equal to the reproductive output, $n_{i}^{*}(x,\;y,\;t)∼\mathrm{Poisson}(n_{i}(x,\;y,\;t)b_{i}(T(x,\;y,\;\;t)))$ [47].

Next, each propagule disperses from its origin (figure 1*b*). Though reproduction occurs within the subpatch level, dispersal occurs at a larger spatial scale (between patches). Therefore, the model pools together all propagules in a patch prior to dispersal, such that the total number of propagules in patch *x* at time *t* is $N_{i}^{*}(x,\;t)=\sum_{y=1}^{W}n_{i}^{*}(x,\;y,\;t)$. We adapt the Laplace dispersal kernel to a bidirectional discrete-space analogue (defined from −∞ to ∞), defining *γ_i_* as the mean absolute distance (in patches) that individuals move from their origin, and let kernel parameter $q_{i}=(\gamma_{i}+1-\sqrt{\gamma_{i}^{2}+1})/\gamma_{i}$. Thus, the probability of a propagule from patch *x* moving to patch *z* is

2.4 $$k(x,\;z)=\left(\frac{q_{i}}{2-q_{i}}\right){\left(1-q_{i}\right)}^{\left|x-z\right|},\;$$

and any propagules that disperse outside the modelled landscape are lost (absorbing boundaries; [39]). All propagules disperse from patch *x* throughout all patches with a multinomial random vector. After arriving at patch *z*, propagules randomly distribute among the *W* subpatches of patch *z*. The resulting number of dispersed propagules in patch *z*, subpatch *y*, at time *t* is $n_{i}^{**}(z,\;y,\;t)$.

Lastly, dispersed propagules compete for limited space and resources within each subpatch, given a location-dependent carrying capacity *K*(*x*, *y*) in each subpatch that remains constant over time (except when modified through management action) (figure 1*c*). The value of *K*(*x*, *y*) varies over space depending on the degree of habitat fragmentation. Density-dependent survival in this model is a variation on lottery competition [48,49] with temperature dependence, with a higher chance of survival around a species thermal optimum *ζ_i_* (equation (2.3)). Altogether, each individual of species *i* has an equal probability of surviving,

2.5 $$p_{i}(x,\;y,\;t)={\left(1+\frac{\sum_{\;j=1}^{S}b_{j}(x,\;y,\;t)\;n_{j}^{**}(x,\;y,\;t)}{b_{i}(x,\;y,\;t)\;K(x,\;y)}\right)}^{-1}.$$

The total number of individuals that survive in patch *x*, subpatch *y*, after competition is a binomial random variable $n_{i}(x,\;y,\;t+1)∼\mathrm{Binomial}(n_{i}^{**}(x,\;y,\;t),\;p(n_{i}^{**}(x,\;y,\;t)))$ [47].

### (c) Management interventions

We simulated six types of management strategies*.* Four of these strategies involved increasing the habitat quality in particular locations by modifying the carrying capacity of those locations. To keep these strategies ecologically comparable, we increased the total carrying capacity by an amount defined as the ‘total area restored’, *E*. We let *K*_u_(*x*, *y*) be the unmanaged carrying capacity of patch *x*, subpatch *y*, and *K*_m_(*x*, *y*) be the carrying capacity after management. Then the total area restored is $E=\sum_{x=1}^{L}\sum_{y=1}^{W}\left(K_{m}(x,\;y)-K_{u}(x,\;y)\right)$.

With the ‘restore all’ strategy, we increased the carrying capacity in all subpatches evenly by *E*/*LW* to represent an equivalent increase in habitat quality in all locations. With the ‘corridor establishment’ strategy, we increased the carrying capacity in all locations that were below a threshold carrying capacity and raised the minimum carrying capacity for all subpatches to that threshold. Therefore, all locations in between high-quality habitats increased in habitat quality to represent increased suitability for species to move through this space. We numerically adjusted this threshold until the total area was *E*. With the ‘stepping-stone’ strategy, we first identified all locations below a threshold. For each region with multiple patches below this threshold, we raised the carrying capacity for all subpatches in the middle 50% quantile of the gap but left the outer 25% quantiles at initial values. We adjusted this threshold until the total area restored was *E*. These intermediate locations of increased quality then might serve as ‘stepping stones’ between higher-quality habitat. With the ‘reinforce’ strategy, we increased the carrying capacity of all subpatches that were above a threshold, adjusting until the total area was *E*. Therefore, high-quality locations further increased in quality to reinforce their utility to species.

Following Backus & Baskett [39], we simulated assisted migration by relocating species when the total metapopulation of a species fell below a threshold of *η* individuals (figure 1*f*). After the population of a species *i* fell below *η*, we relocated a fraction of the population *ϕ* to a location with a temperature approximately equivalent the species thermal optimum *ζ_i_* in the future. To find this, we identified all locations with temperatures $\bar{T}(x,\;t)\leq \zeta_{i}+0.2$. To avoid relocating a species into an area with low habitat quality, we only relocated the population into locations that fitted the above specifications with *K*(*x*, *y*) > 5. We spread individuals between all subpatches within five patches (two on either side of the target location). After relocating a population, we did not relocate that species again for *α* = 5 years to avoid relocating a population recovering from a previous relocation. We used parameter values from Backus & Baskett [39], relocating *ϕ* = 0.55 of the total population during assisted migration, and set conditions such that only *μ* = 0.8 survived relocation (table 1). To limit assisted migration (to be somewhat comparable to habitat quality modification strategies), we only simulated relocations until we reached a maximum limit of *F* relocacations.

**Table 1.** Definitions of the symbols used in the model.

parameter	symbol	value(s)	unit
total no. species in pre-initialized community	*Ω*	64	species
dispersal distance of species *i*	*γ_i_*	lognormal; mean = 2.5, s.d. = 2.5	patches
thermal optimum of species *i*	*ζ_i_*	uniform; 9.78 to 30.22	°C
thermal tolerance breadth of species *i*	*σ_i_*	lognormal; mean = 5, s.d. = 5	°C
reproductive strength of species *i*	*ρ_i_*	derived from *σ_i_*	—
skewness constant	*λ*	−2.7	—
fraction of population relocated	*ϕ*	0.55	—
assisted migration survival probability	*μ*	0.8	—
low population threshold	*η*	50 or 75	individuals
cooldown time between relocations	*α*	5	years
total no. patches	*L*	512	patches
no. subpatches per patch	*W*	8	—
subpatch-carrying capacity	*K*(*x*, *y*)	varies with space (average 8.25)	individuals
s.d. in local temperature heterogeneity	*H*	uniform; 0 to 2	°C
s.d. in interannual temporal stochasticity	*S*	uniform; 0 to 1	°C
mean annual temperature change	*τ*	0.04	°C yr^−1^
annual temporal autocorrelation	*κ*	0.767	—
total area restored	*E*	$\frac{1}{8}LW$ to *LW* (by $\frac{1}{8}LW$), *LW* to 8*LW* (by *LW*)	individuals
maximum no. relocations allowed	*F*	1 to 8 (by 1), 8 to 64 (by 8)	relocations

### (d) Numerical implementation

For our simulations, we used parameter values from table 1. We used *L* = 512 patches and with *W* = 8 subpatches (a total of 2^12^ discrete locations). The initial mean temperature across the temperature gradient varied linearly from the poleward edge to the equatorward edge. The annual temporal autocorrelation was *κ*, based on the measured combined global land-surface air and sea-surface water temperature anomalies from 1880 to 1979 [50,51].

We represented patch heterogeneity as a simple sinusoidal wave. We chose this representation over a more realistic fractal neutral landscape, often used to model heterogeneity in habitat quality over space [52], in order to have a consistent and repeatable patch structure with fewer random variables to consider as we focused on other model comparisons. On average, the carrying capacity was a temperature-independent constant *K*(*x*, *y*) = 8.25 per subpatch, so each patch could carry a total of 66 individuals at carrying capacity. In our simulations, we focused on two theoretical arrangements of high- and low-quality areas to represent different types of fragmentation: one with few wide gaps in habitat quality and one with several narrow gaps (figure 1*e*). In each, the outer edges (*x* ≤ 64 and *x* ≥ 465) were at a constant intermediate carrying capacity *K*(*x*, *y*) = 8.25, while the centre (65 ≤ *x* < 464) varied sinusoidally such that

2.6 $$K(x,\;y)=\frac{1}{4}+8\left(1+\sin\left(\frac{(x-64)\pi }{G}\right)\right).$$

In environments with few wide gaps, *G* = 50, such that there were four full sine waves in the central region (spanning roughly 18.5°C of temperature change over space). In environments with several narrow gaps, *G* = 12.5, with 16 full sine waves in the central region.

In each set of simulations, we first generated the environment by randomly selecting the standard deviation of local heterogeneity *H* and environmental stochasticity *S* (table 1). Next, we generated 64 species, selecting unique random values for each species' thermal optima *ζ_i_*, thermal tolerance breadth *σ_i_*, and dispersal distance *γ_i_*. We numerically derived the reproductive strength *ρ_i_*, such that each species had the same overall reproductive potential *B* = 10 when integrating over temperature, emulating a jack-of-all-trades–master-of-none trade-off (i.e. species with wider niche breadth have lower fecundity and competitive ability in a given environment; [53]). To generate the initial distribution and population size for all species in the community, we first placed four individuals from all species in all subpatches. Then, to find stationary-like conditions prior to simulating climate change, we ran the model for 500 time steps with no change in average yearly temperature (*τ* = 0°C yr^−1^), after which extinctions were unlikely (electronic supplementary material, figures S1 and S2). Several species went globally extinct during this initialization phase, such that there was only an average of 22.04 species in environments with few wide gaps and 21.88 in environments with many narrow gaps prior to climate change (electronic supplementary material, figure S3), and the species that did survive represented a narrower set of ecological values (*ζ_i_*, *σ_i_* and *γ_i_*) than the pool of values that we randomly generated for them (electronic supplementary material, figure S4). At the end of this initialization phase, we used the final population sizes for each species in all subpatches as the initial conditions for climate change simulations.

Next, we simulated climate change on these initialized communities by adjusting the average yearly temperature change to *τ* = 0.04°C yr^−1^, roughly based on a ‘business-as-usual’ projected scenario [3,54]. This scenario provides the greatest number of extinctions with which to compare the relative efficacy of the different management strategies, where we expect relative efficacy (the focus of our analysis) to remain consistent across different climate scenarios. For each community, we simulated the models for both 30 and 100 time steps after applying one of several management scenarios and degrees of management effort. In particular, starting at the beginning of the climate change (shift from *τ* = 0°C yr^−1^ to *τ* = 0.04°C yr^−1^), we simulated ‘restore all’, ‘corridor establishment’, ‘stepping-stone’, and ‘reinforce’ management strategies with total area restored values between *E* = (1/8)*LW* and *E* = 8*LW* (with 16 total variations; table 1). Similarly, we simulated two threshold values for assisted migration (*η* = 50 or *η* = 75 individuals) with a maximum number of relocations between *F* = 1 and *F* = 64 (with 16 total variations; table 1). For comparison, we also simulated community dynamics with no management effort (*E* = 0 and *F* = 0).

To evaluate how spatio-temporal heterogeneity affected management outcomes, we compared the number of extinctions prevented for corridor establishment and assisted migration (*η* = 75) under different levels of environmental stochasticity and local heterogeneity. To use comparable scenarios between these strategies, we chose values for *E* and *F* such that both strategies had a similar number of extinctions on average (*E* = 4*WL* for corridors and *F* = 8 for assisted migration). To evaluate which species benefited under the different management strategies, we found the extinction probability for each management action for species in each community that faced a variety of climate limitations: the species with the shortest average dispersal distance, the species with the narrowest thermal tolerance, the species with strongest competition in the poleward and equatorward direction (smallest difference in *ζ_i_* values), and a random species for comparison.

## 3. Results

**Figure 2.**
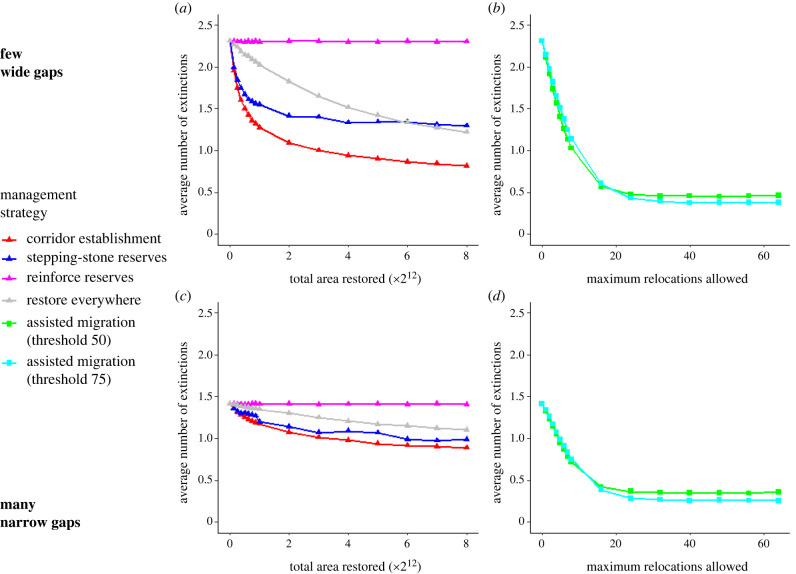
Average number of extinctions after 100 years (*y*-axis) in climate change simulations, depending on management strategy (colour/shape), amount of area restored for restoration-based modification (*a*,*c*) or maximum number of relocations allowed for assisted migration (*b*,*d*; *x*-axis), and environment structure (*a,b*: few wide gaps, *c,d*: many narrow gaps). Each point is the mean of 10 000 simulations.

Habitat corridors, stepping-stone reserves, and restoring all locations reduced the number of species that went extinct during climate change, and each of these strategies reduced extinctions further when restoring a larger total area (figure 2*a,c*). However, the reinforcing strategy had a negligible effect on extinctions. Both corridors and stepping-stones benefited with relatively little area restored, with diminishing returns with higher area restored, while restoring all locations reduced extinctions nearly linearly with increased area restored. On average, corridors reduced the number of extinctions more than all other restoration-based strategies with equivalent area restored. Stepping-stones reduced extinctions similarly to equivalent corridors with little area restored, but corridors were more effective than stepping-stones with higher area restored, especially in environments with fewer, larger gaps.

Assisted migration reduced extinctions on average, even with very few relocation events (figure 2*b,d*). However, increasing the maximum number of relocations above 16–24 did not reduce the average number of extinctions further. At this point, assisted migration prevented more extinctions on average than corridors at the highest area restored value we simulated. Both population thresholds for assisted migration that we simulated (*η* = 50 and *η* = 75) had similar extinction rates with equivalent relocation maximums.

**Figure 3.**
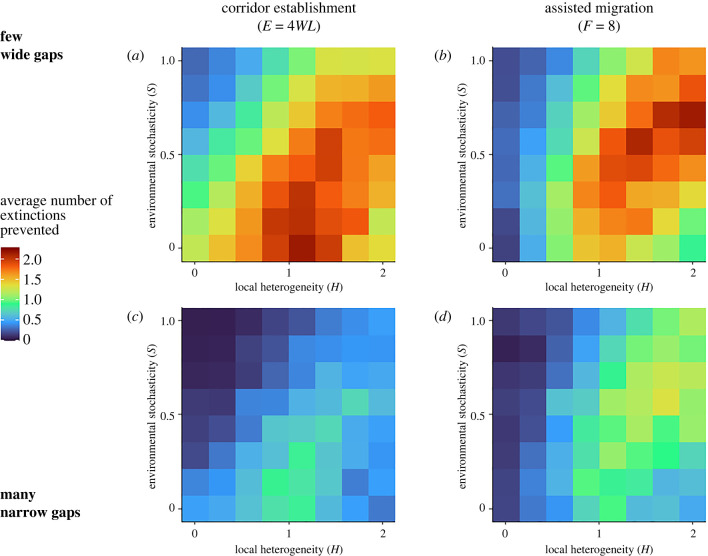
Average number of species prevented from extinction over 100 years (colour) in climate change simulations, depending on local heterogeneity *H* (standard deviation of temperatures per patch, *x*-axis) and environmental stochasticity *S* (standard deviation of interannual variation in temperature, *y*-axis). Each box represents the mean of between 135 and 189 simulations within 8 × 8 quantiles of the range of all simulations. (*a*,*c*) Simulations with corridor establishment; (*b*,*d*) simulations with assisted migration. The total area restored in corridor establishment (*E* = 4*WL*) and the maximum number of relocations in assisted migration (*F* = 8) represent two cases where these two strategies prevent a similar number of extinctions on average for the ‘few wide gaps’ environment, but not when comparing across equivalent levels of *H* and *S*. (*a*,*b*) Simulations of environments with few wide gaps; (*c*,*d*) simulations of environments with several narrow gaps.

Corridors were most effective at preventing extinctions in environments with low environmental stochasticity and moderate local heterogeneity (figure 3*a,c*), while assisted migration was most effective in environments with high heterogeneity and moderate stochasticity (figure 3*b,d*). Neither management strategy was effective at reducing the number of extinctions in environments with low heterogeneity and high stochasticity.

**Figure 4.**
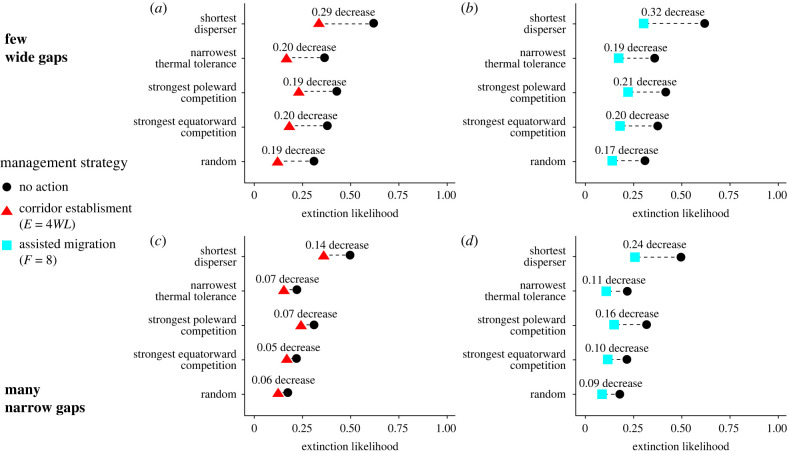
Likelihood that a species went extinct in 100 years (*x*-axis) in our climate change simulations depending on management strategy (colour/shape, with corridors in (*a*,*c*) and assisted migration in (*b*,*d*)), which particular species it was in the community (*y*-axis) and environment type (*a*,*b*: few wide gaps, *c*,*d*: many narrow gaps). The particular species here are the species within the internal region of the environment (65 ≤ *x* < 464) with the shortest dispersal distance *γ_i_*, the species with the narrowest thermal tolerance *σ_i_*, the species in the community with extant neighbouring species community closest to that species' thermal optimum *ζ_i_*, and a randomly chosen species. Each point is the mean of 10 000 simulations.

Randomly chosen species in simulated communities had a lower extinction probability under both corridor and assisted migration strategies, but the shortest-dispersing species in a community disproportionately benefited more than random species (figure 4). Without management action, the shortest-dispersing species had greater than 50% probability of going extinct throughout all variations of our simulations. Both management strategies reduced these extinction probabilities by more than 14% at similar effort levels (*E* = 4*WL* and *F* = 8). Reduction in extinction probability was greater for shortest dispersers than for random species in all scenarios. Other species likely to face extinction during climate change (narrowest thermal tolerance and the smallest difference in thermal optima with neighbouring species on either poleward or equatorward edges) were also less likely to face extinction with either management strategy, but only assisted migration reduced the extinction of these species disproportionately more than random species. Distinguishing the efficacy of assisted migration and corridors for different species and environmental conditions required longer-run (100 time step) simulations, as shorter-run (30 time step) simulations did not have enough extinctions to determine the impact of management interventions on extinction likelihood (2.0–3.5% of species going extinct in 30 time steps versus 18.4–31.6% of species going extinct in 100 time steps; electronic supplementary material, figures S5 and S6).

## 4. Discussion

Most of the simulated management strategies reduced extinction probability under climate change in our simulated communities, and they reduced extinction rapidly with an initial investment in conservation effort. Without climate change, corridors, even when low quality, can facilitate species' movement and long-term persistence in a metacommunity [55,56]. Adding to this, our model suggests that even relatively low-quality corridors between higher-quality areas could reduce extinction during climate change. Because restoring connectivity also increases total habitat area, the effects of increased connectivity and increased area are often confounded [29]. Though many previous studies suggest that habitat reinforcement is often better at protecting species than connectivity restoration [30–32], our results suggest that corridors are likely to be better at increasing the persistence of range-shifting species in the presence of climate change than other methods of connectivity and protection that restore the same amount of area.

Similar to corridors, assisted migration reduced extinctions on average, even with relatively few relocation events in our model. Because many species in the simulated communities face little extinction risk from climate change, focusing relocation on a small number of vulnerable species was able to have a disproportionate effect on community-wide extinctions. Even if only a small number of species are at risk or conservation benefits can be realized by focusing on a few species [57,58], the few species at risk of extinction could require a high investment in management effort on their own. In practice, many conservation translocations are unsuccessful [13,14], so managers might need to relocate a single species several times to increase the overall chance of establishment in the recipient location [39]. Even after successfully establishing a new population, species with weak dispersal ability might continue to lag behind shifting climates and face extinction later. As climate change continues, these conservation-reliant species may depend on repeated direct management actions without increased connectivity [16].

Because we found relatively few extinctions in our nearer-term simulations (30 time steps; electronic supplementary material, figures S5 and S6), the difference in the efficacy of management approaches was negligible, and we required long-run simulations (100 time steps) to show the efficacy of corridors and assisted migration. This potential time lag to observable impact presents a challenge for monitoring to verify anticipated outcomes or adjusting management as needed in an adaptive management approach [59]. However, nearer-term impacts of management action might be evident in cases where optimal climates have already shifted away from species' historical ranges, as has occurred for many species [60,61], and our results suggest that near-term biodiversity conservation management can have long-term benefits for species persistence.

### (a) Types of species benefiting from each management strategy

Adding to the extinction risks caused by fragmentation, many species are at risk of extinction from climate change because of a variety of biological limitations [1,3,28,62]. We found that both corridors and assisted migration were effective at reducing the extinction of species with short dispersal in our model. These species benefited from increased connectivity regardless of the size of low-quality gaps. A previous simulation study showed that longer-dispersing competitors were likely to block shorter-dispersing species from tracking climate change in competitive communities with variable dispersal ability [3]. Without connectivity, short-dispersing species might disperse over patchy landscapes, but low population sizes, low propagule pressure, and strong competition mean that these new populations are unlikely to establish [63,64]. For corals, the species that are likely to have shorter average dispersal range, and likely to benefit from either corridor-like connectivity or assisted migration, are brooding species that release larvae directly from polyps rather than those that broadcast gametes into the water column [65]. Dispersal distance of trees is generally thought to be a function of seed size, tree height, and mode of dispersal [66], where shorter trees that disperse seeds by wind or ballistics are more likely to have shorter dispersal than taller trees that disperse seeds by birds.

In comparison, species with narrow thermal tolerance and strong competition benefited more from assisted migration than restoration-based approaches. In corals, based on a trait-dependent clustering analysis of life-history strategies, those with narrow thermal tolerance (i.e. outside the ‘generalist’ and ‘stress-tolerant’ categories) and likely to experience strong competition (i.e. outside the ‘competitive’ category) fall into a category of ‘weedy’ life histories, which are associated with small colony sizes and reproduction via brooding (where brooding increases reproductive success at low population sizes compared with mass spawning; [67]). Tree species with narrow geographical ranges may have narrow climate tolerance (though see [68]), whereas early successional species may face higher competition [69].

Note that restoration and assisted migration are not dichotomous and can be integrated together in a larger management plan [16]. Most tree species have low dispersal relative to climate change [70], and most corals have narrow climate tolerance relative to climate change [71], so these species could be threatened by climate change for multiple reasons. In these cases, increasing connectivity would benefit most species in the community and assisted migration would benefit those that disproportionately lag behind climate change.

### (b) Environmental characteristics for different management strategies

In our simulations, the optimal management strategy depended on the characteristics of the environment. For example, species in environments with low stochasticity might especially benefit from corridor establishment over assisted migration. Because corridors are relatively small or low-quality compared with the higher-quality areas they connect, the population sizes in those corridors would be relatively small and susceptible to extinction [72]. Lower environmental stochasticity could allow a species to track climate change gradually, alongside several species competing to keep pace with climate change and move through the same limited area of a corridor. In coral reefs, one might identify regions of lower stochasticity through maps of past and projected degree heating weeks, a cumulative stress metric that predicts coral bleaching, which can then serve to inform the designation of reserve networks [36]. In forests, one might preserve larger patches with smaller perimeter-to-area ratio, as edges between forest and fragments experience higher environmental stochasticity and frequency of rare weather events [73,74].

By contrast, we found assisted migration to be particularly effective at reducing extinction in environments with moderate-to-high stochasticity. Because small populations are more likely to face extinction in environments with high environmental stochasticity [72], both donor and recipient populations could face high extinction probability during assisted migration in stochastic environments. However, the benefits of moving a species near its optimal climate likely outweigh the risks of establishment failure on average, especially when planning multiple relocation events and relocating a fraction of a single population each time [39]. Therefore, assisted migration might become an increasingly relevant management tool with increasing environmental variation and extreme events with climate change, such as marine heat waves in coral reefs [75] and extreme droughts or fires affecting forests [76,77]. In our model, assisted migration was also more effective at reducing extinction in environments with higher local heterogeneity. Heterogeneous environments can act as climate refugia [78,79], reducing the velocity of climate change or the negative effects of interannual variation. Because a highly heterogeneous recipient location is more likely to have a suitable microclimate for the relocated population to establish, relocating a population into a refugia-like environment could limit the risk of moving the population into the wrong place at the wrong time. For coral reefs, such local-scale heterogeneity and refugia might arise from fore-reef/back-reef structure, depth gradients and physical structures that drive variability in local upwelling or tidal currents [80]. For forests, high local-scale heterogeneity is often found in areas with steep elevational gradients with similarly steep climate gradients [79].

### (c) Model assumptions

Even though a small amount of restoration or few relocations had large conservation benefits in our simulations, the actual economic and logistical costs of these strategies can be expensive. The total area restored metric does not fully reflect the economic costs of these approaches. To simplify comparison, we assumed that one unit of area restored (increasing the carrying capacity of the community by one individual) is equivalent for all species, regardless of how that area restored is distributed around the simulated environment. Realistically, conservation efforts and cost would vary across species and location [81,82]. A corridor that spreads conservation spending across a wider range of low-quality areas would not be equivalent to a stepping-stone approach that uses the same spending in a smaller, condensed region. Also, considering inherent variation in land and water value or quality [83], it would be difficult to improve the habitat quality of some locations, such as urban coastal waters, beyond a certain point. If the cost of protecting unbroken habitat corridors is prohibitive, ‘land sharing’ approaches that allow conservation and human use to co-occur could enable connectivity [38,84].

Our model also simplifies some important ecological and evolutionary dynamics that might complicate comparisons between restoration and assisted migration-based approaches. In particular, we built our simple competition-based model to represent communities with ranges that are driven by competition and thermal tolerance, but additional biotic and abiotic factors inevitably affect species ranges, range shifts, and the effects of the management approaches modelled here. For example, incorporating trophic interactions or disease dynamics could allow relocated or range-shifted species to become invasive or spread disease, both of which are potential risks of assisted migration [12,85]. Moreover, because complex trophic networks of interacting species can reorganize differentially in response to environmental change depending on their dispersal ability [86], future models might require more detailed food web interactions to predict how fragmented communities respond to climate change. Our discrete-time model also assumes synchronous life cycles for all species, where differences in timing of life cycle events among species can affect competitive outcomes (e.g. priority effects dependent on arrival timing affecting competitive outcomes; [87]) and therefore the dynamics modelled here. Additionally, by representing patch quality as a sinusoidal function over larger spatial scales, we ignored the complexity of realistic heterogeneous patterns. Because natural spatial heterogeneity might be better represented by fractal patterns with lacunarity [52], species might not need to disperse over either large or narrow gaps to keep up with climate change, but a combination of the two on both the larger and smaller scale. We also ignore evolutionary dynamics in this model, which could increase the effectiveness of connectivity-based approaches, as natural dispersal would favour increased gene flow of climate-tolerant genes as species naturally track climate change through corridors [88].

Lastly, we compared the extinction probability of species in our model, but other conservation goals might include maintaining ecosystem function or maintaining biomass for harvesting, among other goals. These alternative goals could favour different management strategies, as the benefits of each strategy are weighed by stakeholders depending on their willingness to engage in assisted migration with its high perceived risk or restoration-based approaches which could involve stakeholders giving up their land or harvesting rights. Further analysis of alternative management strategies to buffer against extinction from climate change and other conservation goals would benefit from a structure-decision making approach that considers the full array of risks, benefits, and uncertainties related to the array of potential stakeholder goals.

## Data Availability

Simulation code, simulation results and code to reproduce the plots in this paper are available at https://github.com/gabackus/comparingManagementStrategies. The data are provided in electronic supplementary material [89].
